# Forecasting Univariate Root-Mean-Square Vibration Sequences: A Benchmark of Statistical, Deep Learning, and Foundation Models on Two Rotating-Machinery Datasets

**DOI:** 10.3390/s26165118

**Published:** 2026-08-12

**Authors:** Thi-Thu-Huong Le, Dawit Shin, Sohaeng Lee, Howon Kim

**Affiliations:** 1 Blockchain Platform Research Center, Pusan National University, Busan 46241, Republic of Korea; imac@pusan.ac.kr; 2 School of Computer Science and Engineering, Pusan National University, Busan 46241, Republic of Korea; sin002277@gmail.com

**Keywords:** bearing vibration, Chronos-Bolt, common-origin evaluation, foundation models, missing-data perturbation, moving-block bootstrap, RMS-feature forecasting, time-series forecasting, TimesFM

## Abstract

Forecasting vibration-derived health indicators is distinct from fault diagnosis and remaining-useful-life estimation, yet controlled comparisons that preserve temporal order, physical scale, and dependence among forecast errors remain limited. This study presents a systematic empirical benchmark for point forecasting univariate root-mean-square (RMS) vibration sequences from two public rotating-machinery datasets with different experimental meanings: “Vibration, Acoustic, Temperature, and Motor Current Dataset of Rotating Machine Under Varying Load Conditions for Fault Diagnosis” (DB1), which provides separately recorded operating and fault conditions, and the “Intelligent Maintenance Systems (IMS) Bearings” record (DB2), from which one run-to-failure recording is used. Twelve methods span naive, statistical, supervised deep-learning, and zero-shot foundation-model families. Every method receives the same 128-step observed context and is evaluated at horizons of 1, 8, and 32 steps on common forecast origins after chronological point-level splitting and training-only scaling. Original-scale errors, per-lead behavior, paired skill, circular moving-block-bootstrap intervals, conditioned cross-regime tests, controlled corruptions, and a desktop central processing unit (CPU) reference workload provide complementary evidence. On DB1 0 Nm Normal, the lowest observed mean absolute error (MAE) is 0.0202 g at the one-step horizon (long short-term memory (LSTM)), 0.0283 g at the eight-step horizon (TimeMixer), and 0.0290 g at the 32-step horizon (inverted Transformer (iTransformer)), although the leading supervised intervals overlap. On DB2 2nd_test, persistence is lowest at the one- and eight-step horizons (0.00788 and 0.01491 dataset acceleration units), while drift is lowest at the 32-step horizon (0.02948); several statistical and zero-shot intervals overlap these leaders. The benchmark combines an explicitly specified waveform-to-target construction, a leakage-safe common-origin design across four model families, dependence-aware inference, and matched robustness and efficiency analyses. The findings show that model value is dataset- and horizon-dependent and that sophisticated forecasters should be judged against strong local baselines under the intended operating context.

## 1. Introduction

Vibration measurements are widely used for rotating-machine condition monitoring because changes in amplitude and frequency content can reflect operating state and mechanical faults [1,2]. Much of the literature concerns fault diagnosis or remaining-useful-life estimation, which require labels or endpoints that differ from direct forecasting of a measured health indicator. The task examined here is narrower: given an observed univariate RMS sequence, predict its next *H* values at a rolling forecast origin. No alarm threshold, failure probability, maintenance action, or remaining-life target is evaluated.

This distinction matters for interpreting model rankings. The DB1 source contains separate recordings acquired under controlled loads and fault conditions [3,4]; it does not provide one continuous normal-to-failure trajectory. The selected DB2 run contains timestamped bearing snapshots and an end-of-test outer-race failure label [5,6], but the authoritative record does not provide an independently verified fault-onset index. Consequently, the study compares forecast accuracy under two specified experimental designs rather than claiming diagnosis, onset detection, or prognosis.

Time-series forecasting methods now span simple extrapolation, statistical models, supervised neural networks, and pretrained zero-shot forecasters. Simple methods remain essential because locally smooth sequences can make persistence or drift difficult to improve upon. The normalized linear model (NLinear) provides a low-complexity learned reference [7]; the patch-based time-series Transformer (PatchTST), inverted Transformer (iTransformer), and TimeMixer represent different neural design choices [8,9,10]; and Chronos and the Time Series Foundation Model (TimesFM) permit zero-shot evaluation [11,12,13]. Comparing these methods is informative only when forecast contexts, target scales, horizons, origins, and uncertainty procedures are explicit.

The study therefore addresses four research questions:

1. How do simple, statistical, supervised, and zero-shot methods compare on the DB1 primary chronological split at all three physical horizons?
2. How do rankings change on DB2 2nd_test when every horizon is evaluated on common forecast origins?
3. What is observed when DB1 models fitted on the Normal recording are conditioned on the past of separately recorded 0 Nm fault conditions?
4. How sensitive are the selected forecasting pipelines to dimension-matched input representations, causal missing-data imputation, raw-waveform noise, and a controlled desktop-CPU workload?

The study makes five specific contributions. It does not propose a new forecasting architecture; instead, it provides a systematic empirical benchmark:

- A systematic waveform-to-RMS benchmark specification is defined for two heterogeneous public sources, with verified units, sampling intervals, subset identities, chronological point boundaries, and training-only preprocessing.
- Twelve methods are compared across naive, statistical, supervised deep-learning, and zero-shot foundation-model families using the same 128-step observed context, three physical horizons, and the intersection of valid forecast origins.
- Original-scale aggregate and per-lead errors are combined with paired circular moving-block-bootstrap inference so that overlapping multi-step targets are not treated as independent evidence.
- The primary benchmark is complemented by a conditioned DB1 cross-regime test and matched nine-method ablations covering alternative scalar representations, repeated causal missingness, and raw-waveform noise before RMS extraction.
- A controlled desktop-CPU workload separates model initialization or loading, training or local refitting, prediction latency, and total process memory; the accompanying public repository provides the core preprocessing and model-execution code.

## 2. Related Work

### 2.1. Machinery Condition Monitoring and Prognostic Targets

Machinery-health studies form a pipeline rather than a single prediction task. The systematic review by Lei et al. separates machinery prognostics into data acquisition, health-indicator construction, health-stage division, and remaining-useful-life (RUL) prediction [14]. Reviews of deep learning for machine health monitoring and machine learning for predictive maintenance likewise cover diagnosis, degradation assessment, prognosis, and maintenance decision support under different label and data requirements [2,15]. This taxonomy is important because low forecast error for a measured indicator does not, by itself, establish diagnostic accuracy, a warning threshold, or an RUL estimate.

Vibration processing is often used to construct compact health indicators from high-rate waveforms. RMS summarizes signal energy on a prescribed segment and yields a lower-rate sequence suitable for monitoring. Its simplicity also limits interpretation: RMS can respond to operating load, sensor placement, and several fault mechanisms, and it discards localized spectral structure. The present work therefore evaluates direct forecasts of an explicitly constructed RMS sequence. It neither learns a diagnostic label nor converts the forecast into a failure endpoint, which distinguishes the task from the bearing RUL study represented by Sun et al. [16].

### 2.2. Direct Forecasting of Vibration-Derived Indicators

Machinery-specific forecasting studies provide useful but narrower precedents. Yuan et al. forecast bearing-vibration RMS characteristic quantities with a gray-model and Markov correction, while Dong and Luo forecast a vibration-derived bearing-degradation process using principal component analysis (PCA) and least-squares support vector machine (LS-SVM) regression [17,18]. Both studies connect an engineered vibration indicator to a particular degradation-oriented application; neither supplies a common-origin comparison across local, supervised, and zero-shot forecasters on heterogeneous public sources. The present study builds on this direct-forecasting strand while retaining the distinction between indicator forecasting and a validated prognostic endpoint.

### 2.3. Forecasting Methods and Benchmark Design

General forecasting surveys organize deep methods by temporal encoding, prediction strategy, distributional output, and the use of local or global training [19,20]. Multi-horizon forecasting is especially relevant here because a single origin produces correlated lead-specific errors, while aggregation can hide degradation at later leads. Large forecasting archives such as the Monash collection improve comparison across domains and emphasize established baselines and multiple error measures [21]. However, such archives are not designed around high-rate vibration waveforms, waveform-to-indicator traceability, or physically interpreted horizons for machinery condition signals.

Autoregressive integrated moving average (ARIMA) and Theta remain established local univariate references [22,23], and mean, persistence, and drift expose whether a learned method adds value beyond level and local trend. LSTM provides gated recurrence [24], whereas NLinear tests whether a single normalized linear projection is sufficient [7]. PatchTST processes temporal patches, iTransformer embeds each variate as a token, and TimeMixer mixes multiple temporal scales [8,9,10]. Because the present task has one variate, iTransformer’s cross-variate attention reduces to a one-token case; its result describes the implemented univariate adaptation, not the full multivariate capability of the original architecture.

### 2.4. Transformers and Time-Series Foundation Models

The Transformer survey by Wen et al. classifies time-series applications into forecasting, anomaly detection, and classification and reviews architectural modifications intended to capture temporal structure [25]. Foundation-model research adds pretraining, adaptation, and data-modality choices to that design space [26]. Chronos treats forecasting as probabilistic sequence modeling, while Chronos-Bolt uses patch-based direct multi-step quantile prediction [11]. TimesFM is a decoder-only patched forecaster; the exact TimesFM 2.5 checkpoint used here is identified separately from the original architecture paper [12,13]. These pretrained models can be evaluated without target-dataset parameter fitting, but their external pretraining means that a zero-shot comparison is not a controlled architecture experiment against models trained only on the target sequence.

### 2.5. Positioning of the Present Study

Table 1 summarizes how the present evaluation relates to the principal review and benchmarking strands. Machinery-health reviews explain the broader diagnostic and prognostic pipeline; direct machinery-forecasting studies address particular vibration-derived indicators; forecasting surveys and archives organize methods and datasets at a general-domain level; Transformer and foundation-model surveys describe rapidly evolving architectures and pretraining strategies. The methodological intersection addressed here is narrower: original-scale, multi-horizon forecasting of an explicitly derived vibration RMS sequence, with identical origins, lead-specific errors, dependence-aware uncertainty, conditioned regime tests, controlled corruptions, and resource accounting applied to statistical, target-trained, and zero-shot models.

## 3. Data and Forecasting Problem

### 3.1. Forecast Definition

Let $x_{t}$ be one RMS value at timeline index *t*. For lookback L=128 and forecast origin *o*, every method receives

(1) $$x_{o-L:o-1}=\left(x_{o-L},\ldots ,x_{o-1}\right)$$

and predicts

(2) $${\hat{x}}_{o:o+H-1}=\left({\hat{x}}_{o},\ldots ,{\hat{x}}_{o+H-1}\right),\;H\in \left\{1,8,32\right\}.$$

At each rolling origin, the context contains observations available up to that origin; predictions from an earlier origin are not recursively inserted into a later context. Supervised models are trained separately for each horizon and directly emit *H* values.

### 3.2. DB1: Controlled Rotating-Machine Recordings

DB1 is the version-6 Mendeley Data record “Vibration, Acoustic, Temperature, and Motor Current Dataset of Rotating Machine Under Varying Load Conditions for Fault Diagnosis” (digital object identifier (DOI): 10.17632/ztmf3m7h5x.6; Creative Commons Attribution 4.0 (CC BY 4.0)) [3,4]. The experiment uses only the vibration channel x_direction_housing_A, measured in gravitational acceleration (*g*), sampled at 25.6 kHz. The primary recording is 0Nm_Normal. Cross-condition analysis uses the 14 separately recorded 0 Nm ball-pass frequency inner-race (BPFI), ball-pass frequency outer-race (BPFO), misalignment, and unbalance files. DB1 therefore tests within-record forecasting and conditioned forecasting across observed regimes; it does not test prediction of an unseen fault before onset.

Each DB1 waveform is filtered with a fourth-order 10–8000 Hz Butterworth bandpass and zero-phase application. RMS is then computed in 2560-sample windows with a 1280-sample stride:

(3) $$x_{t}=\sqrt{\frac{1}{2560}\sum_{i=1}^{2560}s_{t,i}^{2}}.$$

The 0.05 s stride yields 23,999 RMS steps for the 120 s Normal file and 4799 steps for each 60 s fault file.

### 3.3. DB2: IMS 2nd_Test

DB2 is the National Aeronautics and Space Administration (NASA) Open Data “IMS Bearings” record, identifier https://data.nasa.gov/dataset/ims-bearings, accessed on 17 May 2026 [5]. The evaluated subset is 2nd_test: 984 one-second files recorded every 600 s from 12 February to 19 February 2004. Each file contains 20,480 samples at 20 kHz and four columns, one for each bearing in this run. Bearing 1 (column 0) is selected. The local readme reports an outer-race failure for Bearing 1 at the end of the test; it does not publish a fault-onset timestamp. All 984 snapshots, including the final two low-RMS snapshots, are retained.

After the same 10–8000 Hz bandpass, one whole-snapshot RMS value is computed from each file:

(4) $$x_{t}=\sqrt{\frac{1}{20480}\sum_{i=1}^{20480}s_{t,i}^{2}}.$$

**Figure 1:**
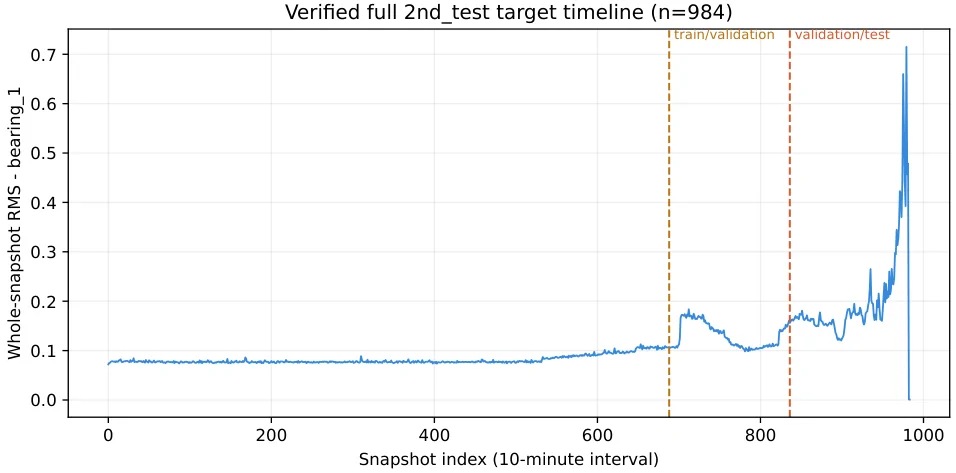
Verified DB2 target timeline containing all 984 Bearing 1 snapshots. Vertical boundaries show the train, validation, and test partitions. No fault-onset marker is drawn because the authoritative record does not provide one.

The authoritative record does not specify a physical acceleration unit, so DB2 scale-dependent errors are labeled “dataset acceleration unit” rather than *g* (see Table 2 and Figure 1).

## 4. Methods

**Figure 2:**
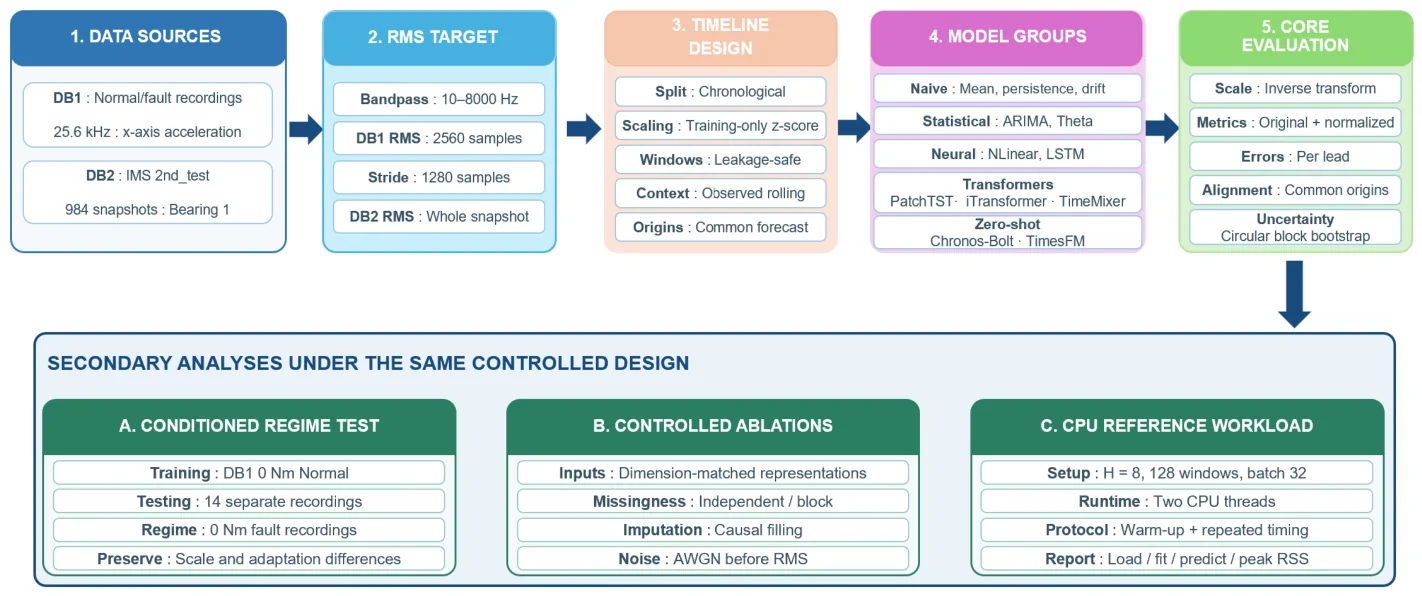
Evaluation framework for univariate RMS vibration forecasting. The five upper stages define the common data-to-evidence path. Conditioned regime testing, controlled ablations, and the CPU reference workload reuse the resulting forecast artifacts or the same target-construction rules.

Figure 2 presents the complete evaluation logic from the two waveform sources to the primary benchmark and the three secondary analyses. The framework standardizes target construction, temporal information, forecast origins, and evidence reporting; it is not introduced as a forecasting architecture.

### 4.1. Leakage-Safe Timeline Construction

For the systematic empirical benchmark, the evaluation workflow performs the following operations in order: (1) build the chronological RMS timeline; (2) define point-level train, validation, and test boundaries; (3) fit a z-score scaler only on the training interval; (4) transform the full timeline with those training statistics; and (5) construct windows whose targets lie entirely inside their assigned partition. Validation and test contexts may contain observed history immediately before the target partition, but target intervals are disjoint. Automated assertions check origin ordering, target containment, timestamp regularity, scaler scope, and cross-horizon origin intersection. No prediction clipping is applied. All scale-dependent metrics are computed after the target scaler is inverted.

For DB1 cross-condition analysis, supervised parameters are learned from the Normal train/validation intervals and the Normal scaler is retained. At fault-test time, every method receives the same 128 observed values from the fault recording. Parameter-free, local statistical, supervised, and pretrained methods consequently have different adaptation mechanisms; results are reported by method rather than interpreted as a pure architecture comparison.

### 4.2. Forecasting Methods

Three parameter-free references are used: the context mean, persistence, and linear drift from the first to the last context value. Theta is fitted locally with period 1, no deseasonalization, and maximum-likelihood estimation [23]. ARIMA order is selected once from the final 512 training points by the Akaike information criterion (AIC) over p,q≤5, d≤1, and p+q≤5; the selected order is then refitted on each 128-step test context. Integrated models use no trend, non-integrated models use a constant, and a failed fit raises an error rather than silently falling back.

The five supervised methods are NLinear, LSTM, PatchTST, iTransformer, and TimeMixer. NLinear subtracts the last context value, applies a single linear projection, and adds the level back. LSTM has two 128-unit layers, dropout 0.1, and a direct horizon head. PatchTST uses patch length 16, stride 8, $d_{\mathrm{model}}=128$, three encoder layers, eight heads, rectified linear unit (ReLU) activation, dropout 0.1, and no reversible instance normalization (RevIN). The univariate iTransformer adaptation uses the same $d_{\mathrm{model}}$, layers, heads, activation, dropout, and no RevIN; its cross-variate attention has one token. The compact TimeMixer adaptation uses $d_{\mathrm{model}}=64$, two past-decomposable-mixing (PDM) blocks, scales {1,2,4}, moving-average kernel 3, Gaussian error linear unit (GELU) activation, and dropout 0.1. These compact implementations are named explicitly to avoid treating them as exact replications of every setting in the source papers.

Chronos-Bolt-small is loaded as amazon/chronos-bolt-small (47,718,016 parameters). It produces patch-based direct multi-step quantiles; the q=0.5 output is used as the point forecast [27]. TimesFM is loaded as google/timesfm-2.5-200m-transformers (231,289,280 parameters) [13]. The checkpoint uses input patches of 32, an output horizon of 128, 20 layers, hidden size 1280, 16 heads, and mean predictions as its point forecast. TimesFM internal context scaling is retained in addition to the common external training-timeline z-score.

The saved model specifications record chronos-forecasting 2.3.1 and Transformers 5.4.0 for DB1, and 2.3.0 and 5.12.1 for DB2. Identical checkpoint identifiers are used for both data sources.

### 4.3. Training and Checkpoint Selection

Each supervised horizon-specific model minimizes mean squared error (MSE) for at most 100 epochs with batch size 32, gradient clipping at 1.0, validation-MSE monitoring, patience 10, and restoration of the lowest-validation-MSE checkpoint. A ReduceLROnPlateau scheduler uses factor 0.5, patience 5, and minimum learning rate $10^{-6}$. LSTM, NLinear, and TimeMixer use learning rate $10^{-3}$; PatchTST and iTransformer use $10^{-4}$. The final activation is the identity. Supervised results use seeds 7, 42, and 123; deterministic methods use seed 7 as a bookkeeping identifier. Three seeds provide limited evidence about optimization variability, so temporal block-bootstrap intervals are also reported and fine-grained ranking claims are avoided.

Training and primary inference use 32-bit floating-point (FP32) precision and select a Compute Unified Device Architecture (CUDA)-enabled graphics processing unit (GPU) when available, otherwise the CPU. The separate efficiency experiment described below is CPU-only. This distinction prevents hardware used to accelerate fitting from being conflated with the controlled CPU reference workload.

### 4.4. Metrics, Common Origins, and Uncertainty

For inverse-transformed targets $y_{oh}$ and predictions ${\hat{y}}_{oh}$, the principal metrics are mean absolute error (MAE), root mean square error (RMSE), and symmetric mean absolute percentage error (sMAPE):

(5) $$\begin{array}{cc} \mathrm{MAE} & =\frac{1}{NH}\sum_{o=1}^{N}\sum_{h=1}^{H}\left|{\hat{y}}_{oh}-y_{oh}\right|, \end{array}$$

(6) $$\begin{array}{cc} \mathrm{RMSE} & =\sqrt{\frac{1}{NH}\sum_{o=1}^{N}\sum_{h=1}^{H}{\left({\hat{y}}_{oh}-y_{oh}\right)}^{2}}, \end{array}$$

(7) $$\begin{array}{cc} \mathrm{sMAPE} & =\frac{100}{NH}\sum_{o,h}\frac{|{\hat{y}}_{oh}-y_{oh}|}{\max((|{\hat{y}}_{oh}\left|+\right|y_{oh}\left|\right)/2,10^{-12})}. \end{array}$$

Mean absolute scaled error (MASE) divides MAE by the mean absolute first difference of the original, non-windowed training timeline. Normalized root mean square error (NRMSE) divides RMSE by the test-target range and is therefore a retrospective normalized metric. $R^{2}$ is calculated over the flattened origin–lead pairs; per-lead metrics are additionally reported so this aggregation is visible.

All horizons use the intersection of valid test origins. An origin–lead pair is the sampling unit for point estimates, so a target timestamp may contribute to more than one rolling-origin forecast. Dependence is addressed for interval estimation rather than ignored: each circular moving-block-bootstrap resample draws contiguous blocks of origins and retains all *H* lead errors belonging to every selected origin. Per-lead files report h=1,…,H. For each model and horizon, a 95% percentile interval is obtained from 1000 such resamples over ordered forecast origins, seed 12,345, with block length $\max(H,\left\lceil N^{1/3}\right\rceil )$. Supervised per-origin errors are averaged across the three seeds before temporal resampling. Paired skill and MAE-difference intervals against persistence use identical resampled blocks [28].

### 4.5. Controlled Ablations and CPU Reference Workload

Ablations use H=8 and nine methods: persistence, drift, NLinear, LSTM, PatchTST, iTransformer, TimeMixer, Chronos-Bolt, and TimesFM. A1 compares dimension-matched scalar input timelines: RMS, the center sample of each filtered waveform segment/snapshot, and spectral centroid. Each representation is tested with and without training-only Gaussian jitter of standard deviation 0.05 on the standardized input. The target remains clean RMS.

A2 corrupts test contexts after standardization using independent point dropout and contiguous blocks of length 4 or 16 at nominal rates of 5%, 10%, and 20%. Missing values are filled strictly left-to-right; an unavailable initial value is set to zero, the training mean on the standardized scale. Five mask realizations are used, and achieved rates are recorded. The result therefore describes the complete corruption–causal-imputation–forecasting pipeline.

A3 adds additive white Gaussian noise (AWGN) to each raw test waveform before bandpass filtering and RMS extraction at signal-to-noise ratios (SNRs) of 30, 20, and 10 decibels (dB). Clean RMS targets and the clean training scaler are unchanged, and five noise realizations are used. The design tests raw-waveform perturbation, but it does not reproduce clipping, drift, packet loss, or all sensor failure modes.

The CPU reference workload uses one Intel Core i7-10700 desktop under Windows 11, OpenBLAS 0.3.33, two CPU threads, 128 windows, context 128, H=8, batch size 32, two warm-up runs, and five measured repeats in a fresh process for every method. Load, training/refit, and prediction are separated. Latency is reported as aggregate-workload throughput divided by 128 windows; the millisecond values for fast baselines therefore retain microsecond-level precision. Memory is total peak resident set size (RSS), including the process baseline and model weights, sampled every 5 ms with psutil.Process().memory_info().rss. Medians and interquartile ranges (IQRs) are reported. No CPU-governor control or separate edge device is evaluated.

## 5. Results

### 5.1. DB1 Primary Chronological Split

**Figure 3:**
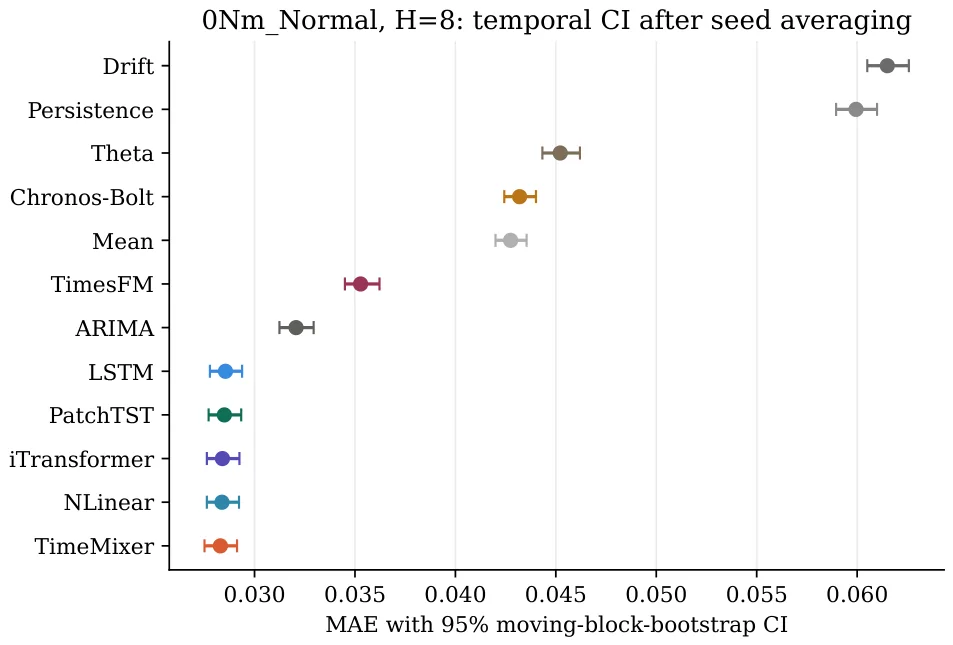
DB1 MAE estimates and temporal moving-block-bootstrap intervals on the original *g* scale.

Table 3 gives all 12 methods and three horizons on the 3569 common test origins. LSTM has the lowest observed mean MAE at H=1 (0.0202 g; 95% confidence interval (CI): 0.0197–0.0208), TimeMixer at H=8 (0.0283 g; 0.0275–0.0291), and iTransformer at H=32 (0.0290 g; 0.0283–0.0298). NLinear, LSTM, PatchTST, iTransformer, and TimeMixer are closely grouped, and their uncertainty intervals overlap. Across-seed variation for supervised models is summarized by the standard deviation (SD). The evidence supports a leading supervised group on this one Normal recording, not a universal ranking among architectures (see Figure 3).

**Figure 4:**
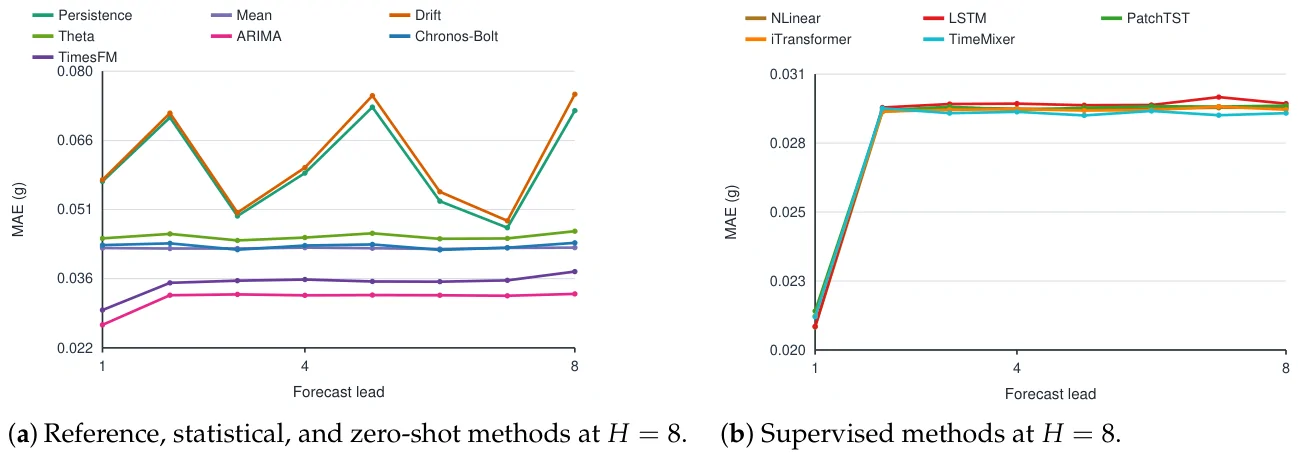

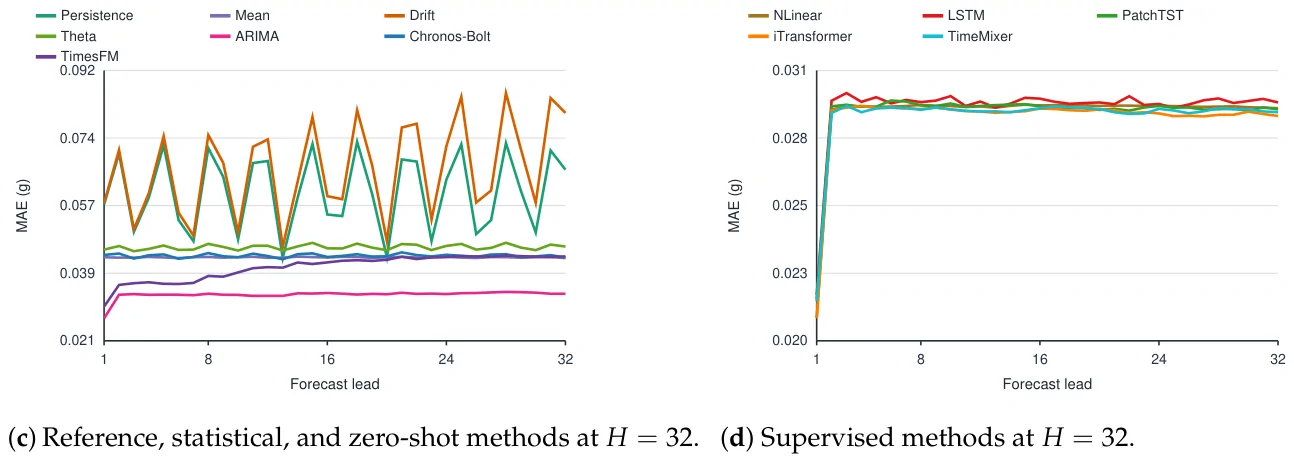
DB1 per-lead MAE on the common forecast origins. Separating reference and supervised methods prevents curves with different error ranges from obscuring one another. Supervised curves are seed means (Table 4).

Figure 4 reports every lead for H=8 and H=32. Alternating persistence and drift errors are observed at successive leads. The supervised curves rise from lead 1 and then remain tightly grouped; this pattern is descriptive and no stationarity mechanism was tested.

### 5.2. DB1 Conditioned Forecasting Across 0 Nm Recordings

Table 5 summarizes equal-condition-weighted MAE over 14 separately recorded fault conditions. TimesFM and ARIMA have the lowest descriptive averages among several horizons, whereas the selected supervised deep models have much larger average errors under the Normal-fitted scaler. NLinear is markedly closer to the statistical and zero-shot group than the other supervised implementations. These comparisons combine distinct adaptation mechanisms and signal scales. Lower absolute error in one condition is not evidence of stronger transfer when the condition itself has a smaller amplitude range.

**Figure 5:**
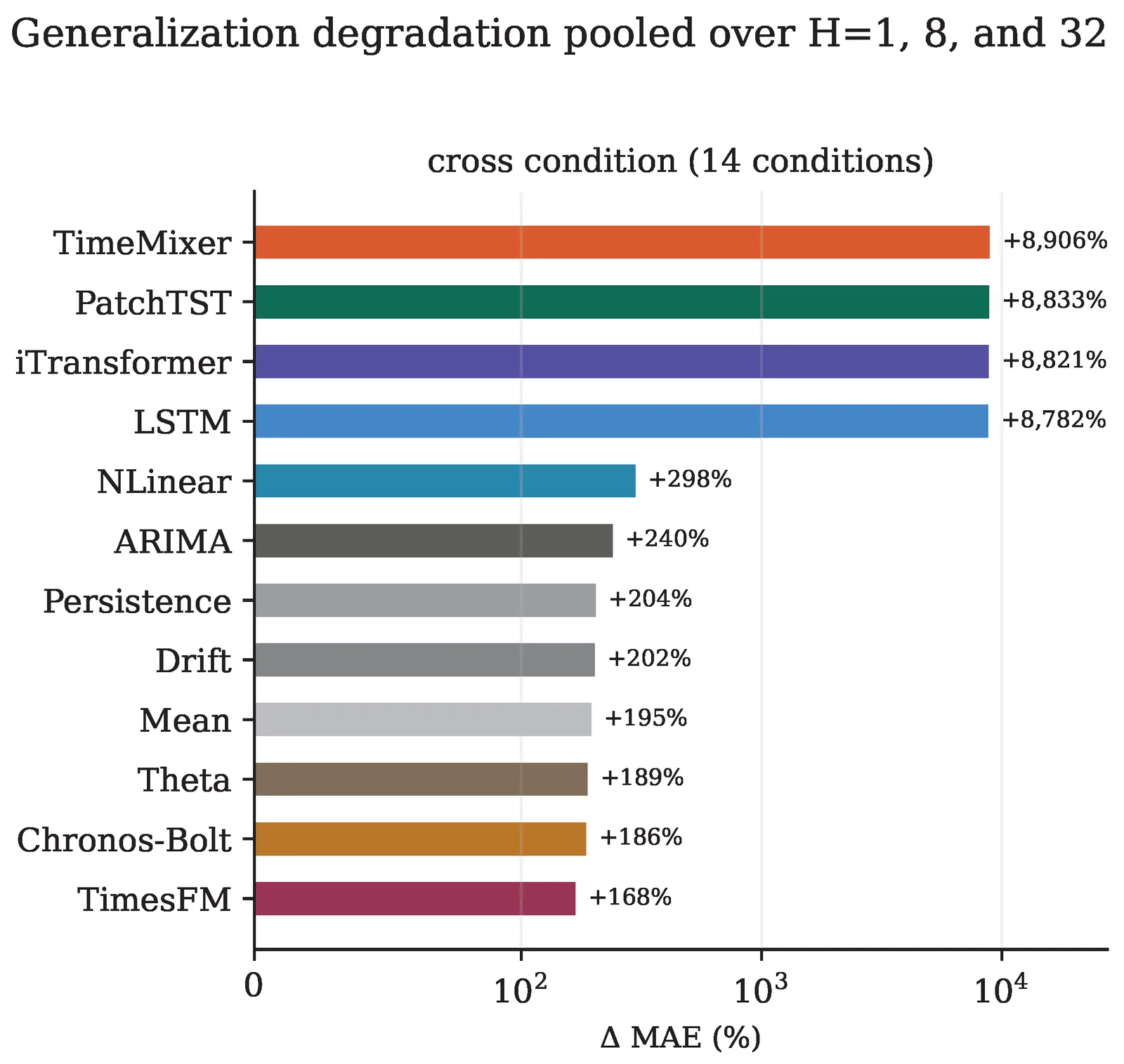
DB1 condition-specific comparison for the 14 held-out 0 Nm fault recordings. Relative quantities are calculated within each condition to reduce, but not eliminate, scale confounding; paired-interval counts are summarized in Table 5.

Figure 5 plots condition-specific relative performance rather than an unweighted radar area, while Table 5 states the equal-condition aggregation and paired-interval counts. This experiment is termed conditioned forecasting across observed regimes; it does not predict a fault before onset and does not represent all loads.

### 5.3. DB2 2nd_Test

Table 6 uses the same 117 test origins for every horizon. Persistence has the lowest observed mean MAE at H=1 (0.00788; 95% CI 0.00556–0.01056) and H=8 (0.01491; 0.01030–0.01948). Drift has the lowest value at H=32 (0.02948; 0.01642–0.04675). At H=1 and H=8, persistence, drift, Theta, ARIMA, Chronos-Bolt, and TimesFM differ by less than 0.001 dataset acceleration units in mean MAE. Their intervals overlap, so the result is an overlapping leading group rather than superiority of one method.

**Figure 6:**
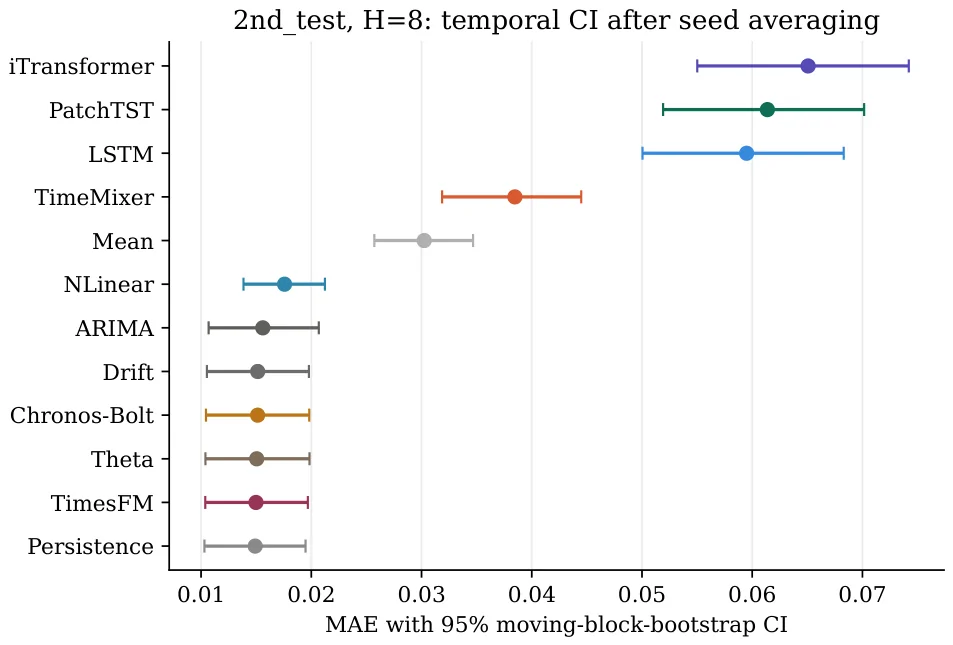
DB 2 MAE estimates and temporal moving-block-bootstrap intervals on 117 common origins. Several simple, statistical, and zero-shot intervals overlap.

**Figure 7:**
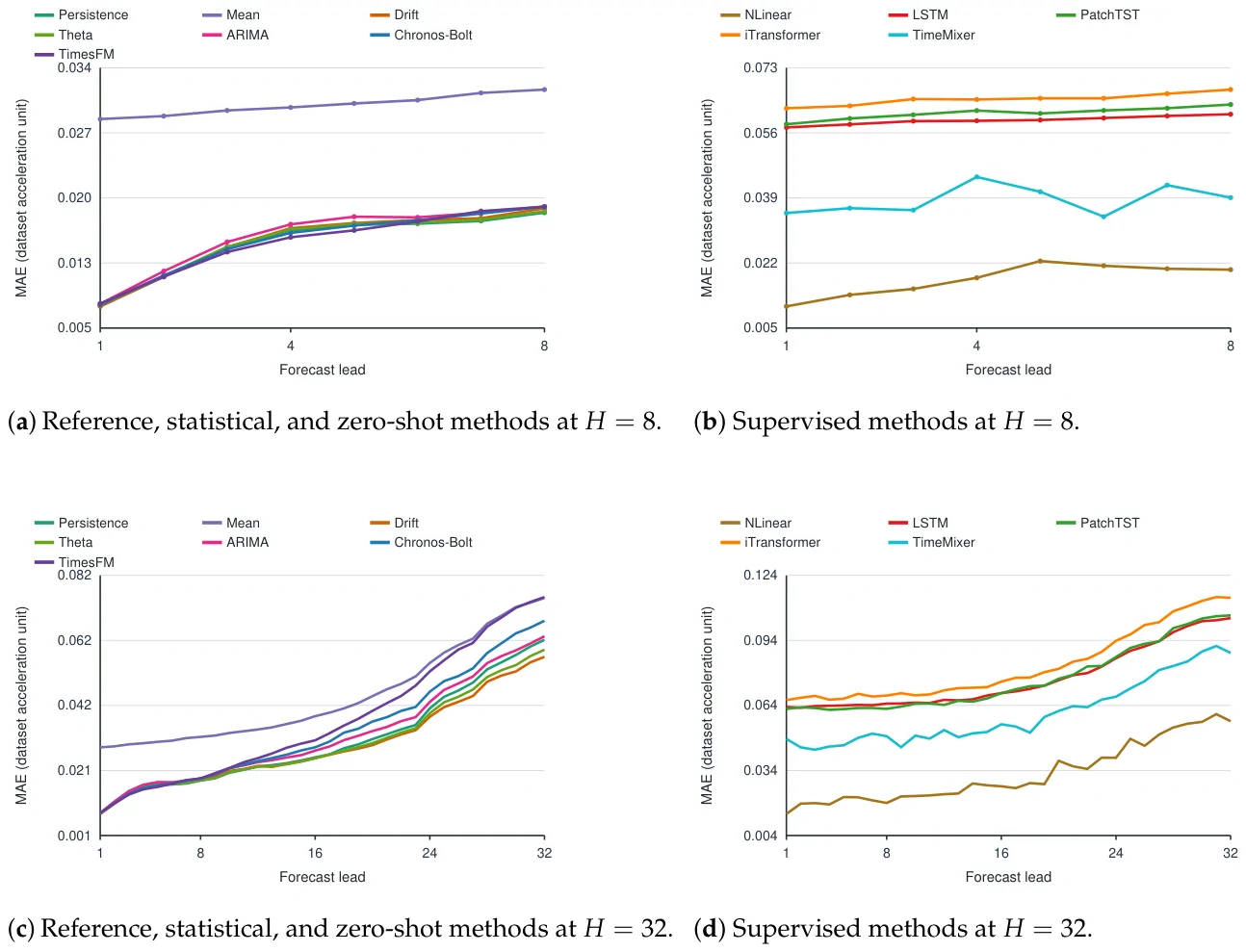
DB2 per-lead MAE on the common forecast origins. Separate panels retain the scale of the leading low-error curves without compressing the supervised results. Supervised curves are seed means.

NLinear is the closest supervised method to this group (MAE 0.01043, 0.01757, and 0.03285). LSTM, PatchTST, the univariate iTransformer adaptation, and TimeMixer have higher errors on this split. Architecture is only one possible contributor; training-range coverage, scaling, compact implementation choices, tuning, and univariate suitability were not isolated. DB2 MASE values are large because the training-only one-step naive scale is 0.00177, so even small original-scale errors are several times that denominator. Low or negative $R^{2}$ values also show that a low MAE ranking does not imply that most test variance is explained (see Table 7 and Figure 6 and Figure 7).

### 5.4. Controlled CPU Efficiency

Table 8 and Table 9 report the same controlled workload separately for the two datasets. On DB2, persistence requires a median 0.000216 ms per window, Chronos-Bolt 2.62 ms with 982.36 MB peak RSS, and TimesFM 19.88 ms with 1699.16 MB peak RSS. Theta and rolling local ARIMA are slower at 24.15 and 58.23 ms per window because they fit locally. Load and training/refit costs are shown separately. DB1 training times are longer for several supervised models because its training timeline is larger; they are not averaged with DB2.

**Figure 8:**
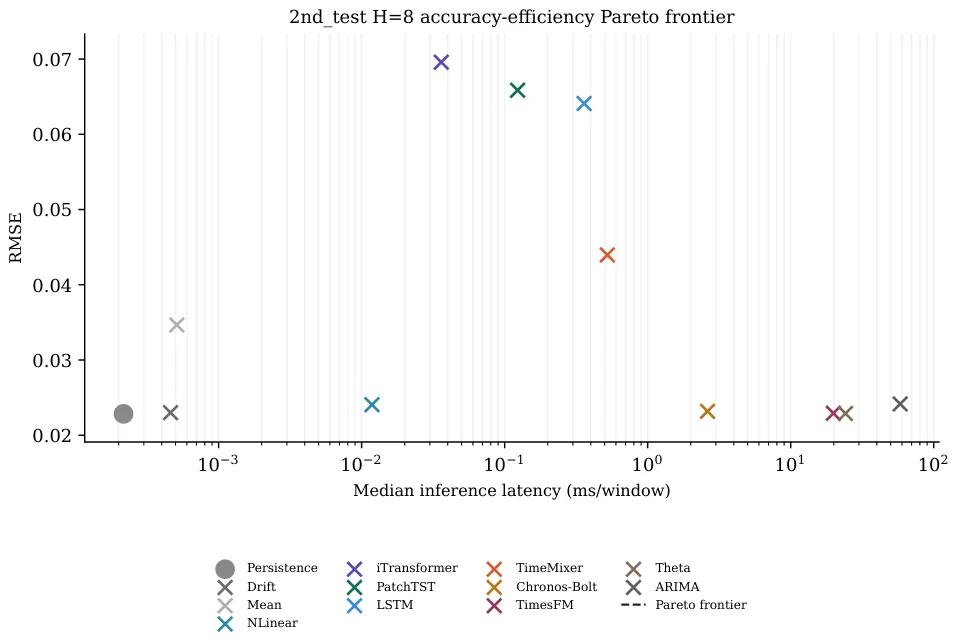
DB2 H=8 accuracy–latency scatter under the controlled desktop-CPU workload. The plotted Pareto frontier is computed from the same verified accuracy and efficiency files used for the tables.

These measurements quantify one desktop configuration. They do not define an acceptable latency or memory threshold for a programmable logic controller (PLC), microcontroller, edge computer, or streaming service. Figure 8 therefore presents measured accuracy–latency positions without prescribing a deployment choice.

### 5.5. Ablation Studies

Table 10 and Table 11 aggregate matched changes over nine methods after first averaging corruption realizations within each model–seed cell. They do not imply that every method responds identically.

For A1, DB1 RMS/no-jitter has mean MAE 0.0330 across cells. Raw center samples increase the mean by 21.26% and spectral centroid by 16.64%; training jitter changes the RMS mean by 0.09%. DB2 RMS/no-jitter has mean MAE 0.06543, compared with 0.11163 for raw center samples and 0.09066 for spectral centroid. The gap between mean and median relative changes on DB2 indicates model heterogeneity. These findings support RMS as a comparatively stable input in this design, not as an essential or universally superior representation.

For A2, the DB1 mean MAE reaches 0.0349 (+6.64%) at the most severe block-16 condition, whose achieved missing rate is 25.19%. The corresponding DB2 mean is 0.06562 (+0.44%). Because causal filling is part of the pipeline, the result cannot be assigned to the forecasting model alone.

**Figure 9:**
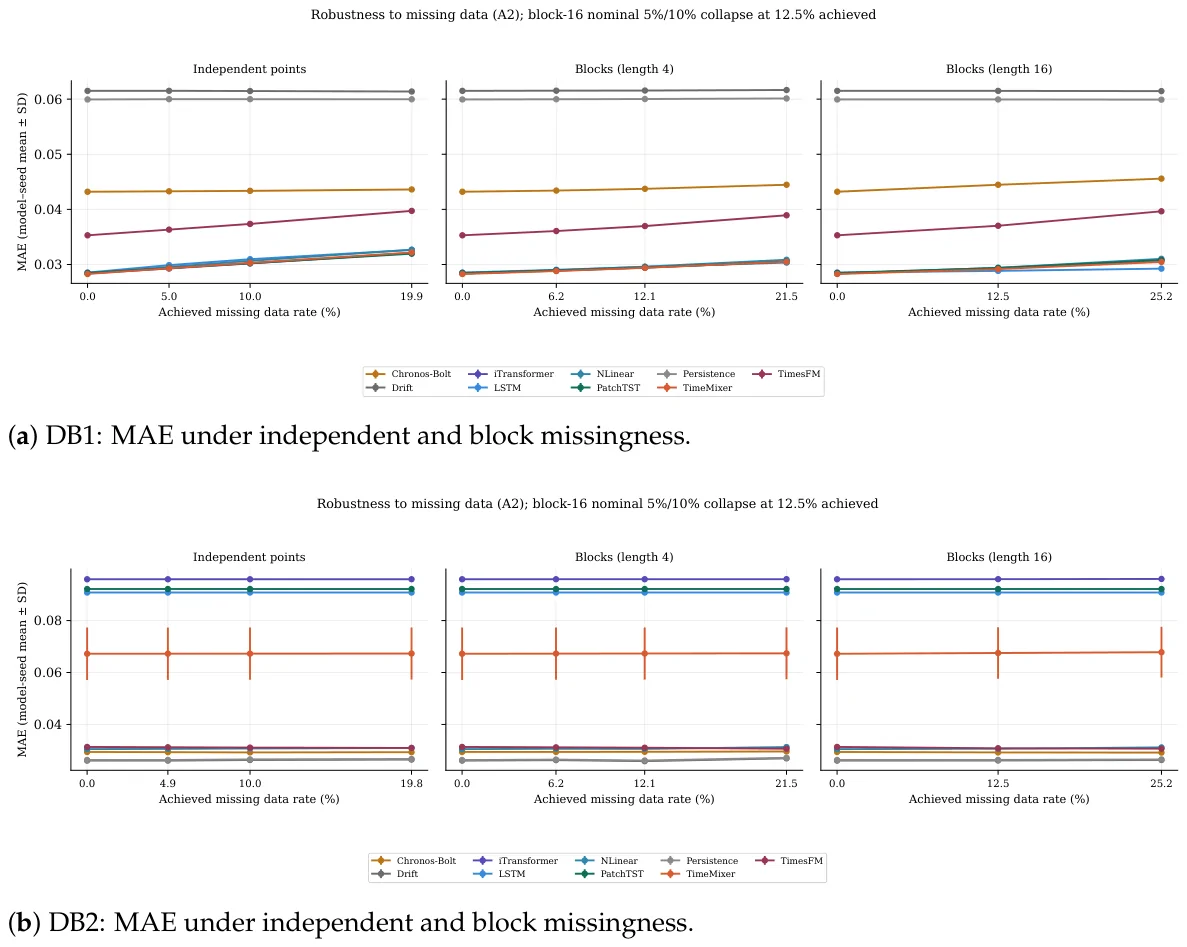
Missing-data ablation with the clean condition, independent masks, block masks, five realizations, and causal forward filling. Variability is calculated from the retained realization-level results.

**Figure 10:**
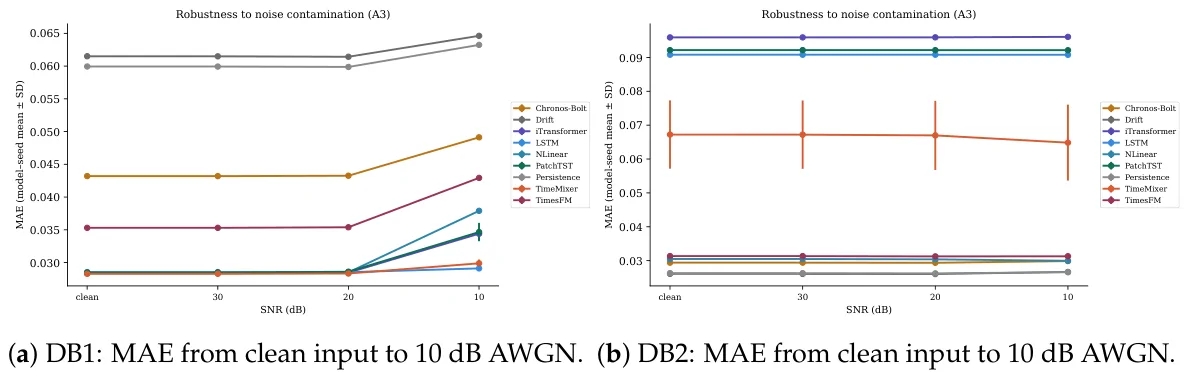
Raw-waveform AWGN ablation before bandpass and RMS extraction. Clean RMS targets and the clean training scaler are retained.

For A3, DB1 is almost unchanged at 30 dB, rises 0.21% at 20 dB, and rises 15.70% at 10 dB. DB2 changes by −0.02%, −0.17%, and −0.61%, respectively, and does not show monotonic degradation in the aggregate. Accordingly, no general noise-tolerance claim is made. A factorial design crossing alternative split regimes with corruption would be required to separate corruption variance from distributional differences (see Figure 9 and Figure 10).

## 6. Discussion

### 6.1. Observed Model-Selection Patterns

The DB1 primary split favors a tightly grouped set of supervised methods, whereas DB2 favors simple local extrapolation and includes the zero-shot checkpoints in a similar low-error group. This contrast is an observation from one Normal recording and one IMS run, not a general rule about stationary and degrading systems. No component analysis establishes why a particular architecture behaves as observed.

The DB1 cross-condition analysis adds a second caution. A method fitted on Normal data may encounter a large shift in level and scale in a separately recorded fault condition, while parameter-free and local models condition directly on the fault history. Absolute MAE, normalized errors, paired skill, and adaptation protocol should therefore be considered together. Averaging condition MAE without this context can reverse an apparent transfer conclusion.

For an analyst working with a new RMS sequence, the defensible takeaway is procedural rather than prescriptive: evaluate persistence, drift, mean, and an established statistical reference before attributing value to a larger model; use chronological splits and common origins; report physical time and original units; and validate latency and alert thresholds on the intended hardware and decision task. The current data do not establish an operational maintenance threshold.

### 6.2. Limitations

The study forecasts a derived signal and does not evaluate fault classification, fault onset, warning lead time, remaining useful life, or maintenance benefit. DB1 primary evidence comes from one 0 Nm Normal recording, with conditioned tests restricted to 14 separate 0 Nm fault recordings; DB2 evidence comes from one run and one bearing channel. Neither source supports broad coverage of gearboxes, pumps, motors, or multiple run-to-failure bearings.

Three optimization seeds give a limited estimate of neural training variability. Moving-block intervals address temporal dependence at forecast origins but do not remove all dependence created by overlapping multi-step targets. NRMSE uses the test range and is retrospective; DB2’s acceleration unit is not physically specified by the authoritative record. The compact PatchTST, iTransformer, and TimeMixer implementations and fixed tuning budget may differ from more extensively tuned variants. External pretraining gives the foundation models access to information not available to target-only supervised models, and DB1/DB2 runs record different library versions.

The ablations are controlled synthetic perturbations. They do not cover drift, clipping, saturation, packet loss, all missingness processes, or multichannel failure. The CPU benchmark is a single desktop measurement without a separately controlled processor governor and is not an industrial-device validation. Broader conclusions require additional machines, loads, bearings, split points, channels, probabilistic calibration, and decision-oriented endpoints.

## 7. Conclusions

This work provides a systematic empirical benchmark of 12 forecasting methods for univariate RMS vibration sequences. Complete DB1 horizons, common forecast origins, low-complexity baselines, and original-scale dependence-aware intervals clarify the observed results. A closely grouped supervised set has the lowest observed errors on the DB1 0 Nm Normal recording. On DB2 2nd_test, persistence or drift has the lowest observed mean MAE at each horizon, with statistical and zero-shot methods frequently overlapping in uncertainty. Conditioned DB1 results, ablations, and desktop-CPU measurements show why scale, adaptation, corruption design, and resource accounting need to accompany headline accuracy.

The main conclusion is limited to the evaluated data: model rankings depend on the exact target construction, split, physical horizon, and available context. Simple baselines provide an essential reference, while larger supervised or pretrained models require evidence of added value under the intended condition and hardware.

## Figures and Tables

**Table 1:** Relationship between representative reviews or benchmarks and the scope of this study.

Study	Literature Role	Primary Task or Scope	Relation to the Present Evaluation
Lei et al. [14]	Systematic machinery-prognostics review	Acquisition, health indicators, stage division, and RUL	Establishes the wider prognostic pipeline; the present endpoint is forecast error for one derived indicator, not RUL.
Zhao et al. [2]	Machine-health-monitoring review	Deep learning for diagnosis and prognosis	Motivates model families but does not provide a common-origin RMS forecasting benchmark.
Carvalho et al. [15]	Predictive-maintenance systematic review	Machine-learning methods and maintenance applications	Emphasizes application-dependent evaluation; the present work isolates a forecasting component without claiming a maintenance decision.
Lim and Zohren [19]; Benidis et al. [20]	Forecasting surveys	Deep architectures and multi-horizon forecasting	Supply the general forecasting taxonomy; vibration target construction and temporal dependence remain application-specific.
Godahewa et al. [21]	General forecasting archive	Diverse domains, baseline methods, and multiple metrics	Supports standardized benchmarks but does not target waveform-derived machinery indicators.
Yuan et al. [17]; Dong and Luo [18]	Direct machinery-indicator forecasting	Bearing-vibration RMS or degradation indicators	Establish direct forecasting precedents, but use application-specific designs rather than a common-origin, multi-family benchmark.
Wen et al. [25]	Transformer survey	Forecasting, anomaly detection, and classification	Contextualizes Transformer variants; this study tests compact univariate implementations under one controlled design.
Liang et al. [26]	Foundation-model survey	Architectures, pretraining, adaptation, and modalities	Contextualizes zero-shot models; this study measures them beside local and target-trained alternatives.
This study	Systematic empirical benchmark	Univariate RMS vibration forecasting at H=1,8,32	Adds physical horizons, common origins, per-lead and paired inference, conditioned regimes, corruptions, and CPU resource accounting.

**Table 2:** Verified target construction and chronological partitions. Window counts follow L=128 and are computed after sequence partitioning.

Property	DB1	DB2
Authoritative source	Mendeley Data v6; DOI 10.17632/ztmf3m7h5x.6	NASA Open Data “IMS Bearings”; no dataset DOI assigned
Evaluated subset	0Nm_Normal; 14 held-out 0 Nm fault recordings for cross-condition analysis	2nd_test; Bearing 1, column 0
Waveform-to-target map	2560-sample RMS, stride 1280 after bandpass	One whole-snapshot RMS from 20,480 samples after bandpass
Target unit and step	*g*; 0.05 s	Dataset acceleration unit; 600 s
Timeline length	23,999 RMS steps for primary file	984 RMS steps
Point boundaries	Train [0, 16,799), validation [16,799, 20,399), test [20,399, 23,999)	Train [0, 688), validation [688, 836), test [836, 984)
Windows, H=1	16,671/3600/3600	560/148/148
Windows, H=8	16,664/3593/3593	553/141/141
Windows, H=32	16,640/3569/3569	529/117/117
Common test origins	3569 for every horizon	117 for every horizon
Physical horizons	0.05, 0.40, and 1.60 s	10 min, 1.33 h, and 5.33 h

**Table 3:** Vibration forecasting results on the primary chronological split (0Nm_Normal; supervised models: mean ± std over 3 seeds; deterministic models: one run). sMAPE is computed on the inverse-transformed RMS scale. MAE/RMSE unit: g; physical horizons: H = 1: 0.05 s, = 8: 0.40 s, H = 32: 1.60 s. **Bold** marks the lowest observed error values and highest observed $R^{2}$ within each horizon.

H	Model	MAE	RMSE	sMAPE	MASE	NRMSE	R^2^
1	ARIMA	0.026	0.033	4.280	0.437	0.095	0.602
	Chronos-Bolt ^†^	0.043	0.054	6.991	0.714	0.154	−0.037
	Drift	0.057	0.069	9.257	0.942	0.198	−0.722
	iTransformer	0.021 ± 0.000	0.027 ± 0.000	3.432 ± 0.022	0.352 ± 0.003	0.078 ± 0.000	0.736 ± 0.003
	LSTM	**0.020 ± 0.000**	**0.026 ± 0.000**	**3.248 ± 0.024**	**0.333 ± 0.002**	**0.074 ± 0.001**	**0.757 ± 0.003**
	Mean	0.043	0.053	6.893	0.704	0.152	−0.009
	NLinear	0.021 ± 0.000	0.026 ± 0.000	3.349 ± 0.006	0.343 ± 0.001	0.076 ± 0.000	0.748 ± 0.001
	PatchTST	0.021 ± 0.000	0.027 ± 0.000	3.386 ± 0.035	0.347 ± 0.004	0.077 ± 0.001	0.743 ± 0.004
	Persistence	0.057	0.069	9.203	0.936	0.197	−0.703
	Theta	0.045	0.055	7.218	0.738	0.159	−0.104
	TimeMixer	0.021 ± 0.000	0.026 ± 0.000	3.356 ± 0.006	0.344 ± 0.001	0.076 ± 0.000	0.748 ± 0.001
	TimesFM ^†^	0.030	0.037	4.792	0.488	0.106	0.506
8	ARIMA	0.032	0.040	5.167	0.528	0.115	0.416
	Chronos-Bolt ^†^	0.043	0.053	6.958	0.711	0.153	−0.031
	Drift	0.061	0.076	9.923	1.012	0.218	−1.093
	iTransformer	0.028 ± 0.000	0.036 ± 0.000	4.559 ± 0.011	0.468 ± 0.001	0.103 ± 0.000	0.538 ± 0.003
	LSTM	0.029 ± 0.000	0.036 ± 0.000	4.585 ± 0.027	0.470 ± 0.003	0.104 ± 0.001	0.529 ± 0.005
	Mean	0.043	0.053	6.887	0.704	0.152	−0.008
	NLinear	0.028 ± 0.000	0.036 ± 0.000	4.556 ± 0.001	0.467 ± 0.000	0.102 ± 0.000	0.539 ± 0.000
	PatchTST	0.029 ± 0.000	0.036 ± 0.000	4.572 ± 0.012	0.469 ± 0.001	0.103 ± 0.000	0.536 ± 0.002
	Persistence	0.060	0.074	9.670	0.987	0.213	−0.989
	Theta	0.045	0.056	7.282	0.744	0.160	−0.123
	TimeMixer	**0.028 ± 0.000**	**0.036 ± 0.000**	**4.542 ± 0.006**	**0.466 ± 0.001**	**0.102 ± 0.000**	**0.541 ± 0.001**
	TimesFM ^†^	0.035	0.044	5.690	0.581	0.127	0.296
32	ARIMA	0.033	0.041	5.288	0.540	0.118	0.392
	Chronos-Bolt ^†^	0.043	0.053	6.960	0.711	0.153	−0.032
	Drift	0.066	0.083	10.713	1.093	0.237	−1.457
	iTransformer	**0.029 ± 0.000**	**0.036 ± 0.000**	**4.653 ± 0.002**	**0.477 ± 0.000**	**0.104 ± 0.000**	**0.523 ± 0.000**
	LSTM	0.029 ± 0.000	0.037 ± 0.000	4.722 ± 0.022	0.484 ± 0.002	0.106 ± 0.000	0.506 ± 0.004
	Mean	0.043	0.053	6.890	0.704	0.152	−0.008
	NLinear	0.029 ± 0.000	0.037 ± 0.000	4.686 ± 0.001	0.481 ± 0.000	0.105 ± 0.000	0.518 ± 0.000
	PatchTST	0.029 ± 0.000	0.037 ± 0.000	4.685 ± 0.009	0.481 ± 0.001	0.105 ± 0.000	0.517 ± 0.002
	Persistence	0.060	0.075	9.715	0.992	0.215	−1.021
	Theta	0.045	0.056	7.322	0.748	0.161	−0.136
	TimeMixer	0.029 ± 0.000	0.036 ± 0.000	4.665 ± 0.002	0.479 ± 0.000	0.105 ± 0.000	0.520 ± 0.001
	TimesFM ^†^	0.040	0.050	6.473	0.661	0.143	0.100

^†^ zero-shot, no training.

**Table 4:** DB1 temporal 95% moving-block-bootstrap confidence intervals for MAE on the primary chronological split. For supervised models, per-origin absolute errors are averaged across the three fixed seeds before temporal resampling; the bootstrap unit is the forecast origin. Seed-to-seed SD is reported separately in Table 3.

H	Model	MAE	95% CI low	95% CI High
1	LSTM	0.0202	0.0197	0.0208
1	NLinear	0.0208	0.0203	0.0214
1	TimeMixer	0.0209	0.0204	0.0214
1	PatchTST	0.0211	0.0206	0.0216
1	iTransformer	0.0214	0.0209	0.0219
1	ARIMA	0.0265	0.0259	0.0272
1	TimesFM ^†^	0.0297	0.0289	0.0304
1	Mean	0.0428	0.0420	0.0436
1	Chronos-Bolt ^†^	0.0434	0.0426	0.0443
1	Theta	0.0448	0.0439	0.0458
1	Persistence	0.0569	0.0558	0.0580
1	Drift	0.0572	0.0561	0.0584
8	TimeMixer	0.0283	0.0275	0.0291
8	NLinear	0.0284	0.0276	0.0292
8	iTransformer	0.0284	0.0276	0.0292
8	PatchTST	0.0285	0.0277	0.0293
8	LSTM	0.0286	0.0278	0.0294
8	ARIMA	0.0321	0.0312	0.0329
8	TimesFM ^†^	0.0353	0.0345	0.0362
8	Mean	0.0427	0.0420	0.0435
8	Chronos-Bolt ^†^	0.0432	0.0424	0.0440
8	Theta	0.0452	0.0443	0.0462
8	Persistence	0.0599	0.0590	0.0610
8	Drift	0.0615	0.0605	0.0626
32	iTransformer	0.0290	0.0283	0.0298
32	TimeMixer	0.0291	0.0283	0.0299
32	NLinear	0.0292	0.0284	0.0300
32	PatchTST	0.0292	0.0285	0.0299
32	LSTM	0.0294	0.0287	0.0302
32	ARIMA	0.0328	0.0320	0.0336
32	TimesFM ^†^	0.0402	0.0395	0.0408
32	Mean	0.0428	0.0421	0.0435
32	Chronos-Bolt ^†^	0.0432	0.0425	0.0439
32	Theta	0.0455	0.0445	0.0463
32	Persistence	0.0602	0.0593	0.0612
32	Drift	0.0664	0.0653	0.0674

^†^ zero-shot, no training.

**Table 5:** Compact cross-condition summary over 14 held-out fault conditions. MAE is an equal-weight descriptive mean over conditions and prescribed seeds. Paired-CI counts pool all condition–horizon–seed tests against persistence: positive means the 95% skill CI is entirely above zero and negative means it is entirely below zero.

Model	MAE H = 1	MAE H = 8	MAE H = 32	Positive Paired CI	Negative Paired CI
Persistence	0.1920	0.1729	0.1734	reference	–
Mean	0.1265	0.1260	0.1261	42/42	0/42
Drift	0.1928	0.1768	0.1893	1/42	41/42
Theta	0.1298	0.1301	0.1314	42/42	0/42
ARIMA	0.0840	0.1086	0.1180	33/42	0/42
NLinear	0.1058	0.1036	0.1026	126/126	0/126
LSTM	2.2176	2.3566	2.3700	45/126	81/126
PatchTST	2.3438	2.3433	2.3488	45/126	81/126
iTransformer	2.3318	2.3444	2.3496	45/126	81/126
TimeMixer	2.3620	2.3258	2.3577	45/126	81/126
Chronos-Bolt ^†^	0.1230	0.1238	0.1248	42/42	0/42
TimesFM ^†^	0.0779	0.0915	0.1125	42/42	0/42

^†^ zero-shot, no training.

**Table 6:** Vibration forecasting results on the primary chronological split (2nd_test; supervised models: mean ± std over 3 seeds; deterministic models: one run). sMAPE is computed on the inverse-transformed RMS scale. MAE/RMSE use the dataset acceleration unit because the authoritative record does not specify a physical acceleration unit. Physical horizons: H = 1: 10.0 min, H = 8: 1.33 h, H = 32: 5.33 h. **Bold** = lowest observed value per horizon.

H	Model	MAE	RMSE	sMAPE	MASE	NRMSE	R^2^
1	ARIMA	0.00825	0.01313	4.641	4.669	0.0905	0.6505
	Chronos-Bolt ^†^	0.00807	0.01301	4.562	4.569	0.0896	0.6570
	Drift	0.00790	0.01251	4.450	4.471	0.0862	0.6826
	iTransformer	0.05764 ± 0.00058	0.06154 ± 0.00049	40.932 ± 0.514	32.625 ± 0.326	0.4240 ± 0.0034	−6.6760 ± 0.1225
	LSTM	0.04597 ± 0.00143	0.05072 ± 0.00139	31.105 ± 1.151	26.018 ± 0.809	0.3494 ± 0.0096	−4.2163 ± 0.2881
	Mean	0.02867	0.03277	18.323	16.228	0.2258	−1.1766
	NLinear	0.01043 ± 0.00103	0.01496 ± 0.00121	6.009 ± 0.671	5.904 ± 0.582	0.1031 ± 0.0083	0.5444 ± 0.0726
	PatchTST	0.05857 ± 0.00078	0.06263 ± 0.00070	41.725 ± 0.688	33.150 ± 0.440	0.4315 ± 0.0048	−6.9505 ± 0.1772
	Persistence	**0.00788**	**0.01249**	**4.442**	**4.460**	**0.0860**	**0.6839**
	Theta	0.00810	0.01264	4.568	4.583	0.0871	0.6762
	TimeMixer	0.03967 ± 0.00433	0.04483 ± 0.00349	26.268 ± 3.367	22.452 ± 2.448	0.3089 ± 0.0241	−3.0896 ± 0.6313
	TimesFM ^†^	0.00819	0.01276	4.663	4.635	0.0879	0.6698
8	ARIMA	0.01560	0.02416	8.967	8.829	0.1665	−0.0173
	Chronos-Bolt ^†^	0.01513	0.02319	8.786	8.564	0.1598	0.0634
	Drift	0.01514	0.02301	8.733	8.567	0.1585	0.0779
	iTransformer	0.06507 ± 0.00044	0.06959 ± 0.00049	46.870 ± 0.401	36.827 ± 0.248	0.4795 ± 0.0034	−7.4373 ± 0.1181
	LSTM	0.05949 ± 0.00116	0.06410 ± 0.00112	41.882 ± 1.014	33.671 ± 0.657	0.4416 ± 0.0077	−6.1599 ± 0.2492
	Mean	0.03024	0.03467	19.120	17.116	0.2388	−1.0936
	NLinear	0.01757 ± 0.00155	0.02406 ± 0.00170	10.426 ± 0.926	9.945 ± 0.880	0.1658 ± 0.0117	−0.0122 ± 0.1451
	PatchTST	0.06137 ± 0.00042	0.06586 ± 0.00039	43.546 ± 0.378	34.737 ± 0.240	0.4538 ± 0.0027	−6.5572 ± 0.0885
	Persistence	**0.01491**	**0.02284**	**8.636**	**8.437**	**0.1574**	**0.0908**
	Theta	0.01505	0.02289	8.698	8.516	0.1577	0.0868
	TimeMixer	0.03847 ± 0.00797	0.04397 ± 0.00774	25.240 ± 5.845	21.773 ± 4.510	0.3030 ± 0.0534	−2.4383 ± 1.1879
	TimesFM ^†^	0.01498	0.02292	8.769	8.478	0.1579	0.0845
32	ARIMA	0.03246	0.06036	16.337	18.373	0.0845	0.0055
	Chronos-Bolt ^†^	0.03411	0.06479	17.222	19.306	0.0907	−0.1462
	Drift	**0.02948**	**0.05707**	**14.716**	**16.686**	**0.0799**	**0.1109**
	iTransformer	0.08260 ± 0.00031	0.10264 ± 0.00029	54.387 ± 0.285	46.749 ± 0.176	0.1437 ± 0.0004	−1.8760 ± 0.0164
	LSTM	0.07657 ± 0.00027	0.09732 ± 0.00025	49.024 ± 0.228	43.338 ± 0.150	0.1362 ± 0.0003	−1.5856 ± 0.0132
	Mean	0.04473	0.06936	24.532	25.315	0.0971	−0.3132
	NLinear	0.03285 ± 0.00085	0.05945 ± 0.00092	16.709 ± 0.494	18.592 ± 0.480	0.0832 ± 0.0013	0.0348 ± 0.0298
	PatchTST	0.07654 ± 0.00076	0.09744 ± 0.00065	48.990 ± 0.663	43.324 ± 0.431	0.1364 ± 0.0009	−1.5923 ± 0.0348
	Persistence	0.03087	0.05992	15.507	17.472	0.0839	0.0198
	Theta	0.03001	0.05834	15.021	16.987	0.0817	0.0707
	TimeMixer	0.06055 ± 0.02548	0.08395 ± 0.02218	37.330 ± 18.531	34.270 ± 14.419	0.1175 ± 0.0310	−1.0133 ± 0.9511
	TimesFM ^†^	0.03738	0.06641	19.354	21.156	0.0930	−0.2042

^†^ zero-shot, no training.

**Table 7:** DB2 temporal 95% moving-block-bootstrap confidence intervals for MAE on the primary chronological split. For supervised models, per-origin absolute errors are averaged across the three fixed seeds before temporal resampling; the bootstrap unit is the forecast origin. Seed-to-seed SD is reported separately in Table 6.

H	Model	MAE	95% CI Low	95% CI High
1	Persistence	0.00788	0.00556	0.01056
1	Drift	0.00790	0.00554	0.01062
1	Chronos-Bolt ^†^	0.00807	0.00584	0.01071
1	Theta	0.00810	0.00575	0.01082
1	TimesFM ^†^	0.00819	0.00594	0.01088
1	ARIMA	0.00825	0.00569	0.01129
1	NLinear	0.01043	0.00814	0.01328
1	Mean	0.02867	0.02378	0.03322
1	TimeMixer	0.03967	0.03283	0.04633
1	LSTM	0.04597	0.03864	0.05278
1	iTransformer	0.05764	0.05016	0.06440
1	PatchTST	0.05857	0.05087	0.06556
8	Persistence	0.01491	0.01030	0.01948
8	TimesFM ^†^	0.01498	0.01038	0.01969
8	Theta	0.01505	0.01039	0.01984
8	Chronos-Bolt ^†^	0.01513	0.01044	0.01982
8	Drift	0.01514	0.01053	0.01978
8	ARIMA	0.01560	0.01068	0.02068
8	NLinear	0.01757	0.01385	0.02124
8	Mean	0.03024	0.02572	0.03468
8	TimeMixer	0.03847	0.03186	0.04449
8	LSTM	0.05949	0.05004	0.06830
8	PatchTST	0.06137	0.05191	0.07015
8	iTransformer	0.06507	0.05501	0.07421
32	Drift	0.02948	0.01642	0.04675
32	Theta	0.03001	0.01644	0.04821
32	Persistence	0.03087	0.01649	0.04984
32	ARIMA	0.03246	0.01765	0.05161
32	NLinear	0.03285	0.02089	0.05085
32	Chronos-Bolt ^†^	0.03411	0.01784	0.05582
32	TimesFM ^†^	0.03738	0.02027	0.05883
32	Mean	0.04473	0.02716	0.06745
32	TimeMixer	0.06055	0.03898	0.08859
32	PatchTST	0.07654	0.05093	0.10759
32	LSTM	0.07657	0.05089	0.10764
32	iTransformer	0.08260	0.05619	0.11505

^†^ zero-shot, no training.

**Table 8:** DB1: Dedicated CPU efficiency benchmark at H = 8 using 128 windows, batch size 32, two warm-up runs, five measured repeats, and two CPU threads in a fresh process per model. Medians and interquartile ranges summarize measured repeats.

Model	Init (s)	Load (s)	Training (s)	Latency Median (ms/Window)	Latency IQR	Peak RSS Median (MB)	Peak RSS IQR
Persistence	0.00	0.00	0.00	0.000275	0.000002	661.43	0.01
Drift	0.00	0.00	0.00	0.000580	0.000241	662.48	0.02
Mean	0.00	0.00	0.00	0.000626	0.000009	661.16	0.00
NLinear	0.00	0.02	56.28	0.012	0.000288	664.79	0.00
iTransformer	0.01	0.03	156.48	0.040	0.003381	669.38	0.00
PatchTST	0.01	0.03	123.08	0.126	0.003408	673.18	0.01
LSTM	0.00	0.02	42.03	0.359	0.011	677.53	0.08
TimeMixer	0.01	0.03	223.88	0.550	0.033	690.14	0.00
Chronos-Bolt ^†^	0.00	2.86	0.00	2.97	0.202	996.24	0.00
TimesFM ^†^	0.00	2.91	0.00	22.25	2.51	1712.34	1.91
Theta	0.00	0.00	0.00	74.33	0.506	664.50	0.02
ARIMA	0.00	0.00	7.96	349.45	4.02	671.00	0.03

^†^ zero-shot, no training.

**Table 9:** DB2: Dedicated CPU efficiency benchmark at H = 8 using 128 windows, batch size 32, two warm-up runs, five measured repeats, and two CPU threads in a fresh process per model. Medians and interquartile ranges summarize measured repeats.

Model	Init (s)	Load (s)	Training (s)	Latency median (ms/Window)	Latency IQR	Peak RSS Median (MB)	Peak RSS IQR
Persistence	0.00	0.00	0.00	0.000216	0.000003	647.58	0.00
Drift	0.00	0.00	0.00	0.000461	0.000023	647.66	0.00
Mean	0.00	0.00	0.00	0.000510	0.000045	648.27	0.00
NLinear	0.00	0.00	0.24	0.012	0.000289	651.18	0.02
iTransformer	0.01	0.01	5.64	0.036	0.001495	655.20	0.00
PatchTST	0.01	0.01	9.86	0.123	0.002941	657.82	0.00
LSTM	0.00	0.01	14.81	0.359	0.002361	663.03	0.01
TimeMixer	0.01	0.01	15.83	0.522	0.066	675.84	0.00
Chronos-Bolt ^†^	0.00	2.69	0.00	2.62	0.077	982.36	0.00
TimesFM ^†^	0.00	2.66	0.00	19.88	0.083	1699.16	1.90
Theta	0.00	0.00	0.00	24.15	0.201	651.89	0.01
ARIMA	0.00	0.00	8.06	58.23	1.66	657.80	0.00

^†^ zero-shot, no training.

**Table 10:** DB1 compact ablation summary (vibration, H = 8). Nine forecasting methods are included. Corruption realizations are averaged within each model–seed cell before equal-weight aggregation across cells. Relative changes use RMS/no augmentation (A1) or clean input (A2–A3) as matched baselines. A2 reports the achieved rate; the nominal 5% and 10% block-16 cases are identical one-block masks and are collapsed.

Ablation	Setting	Nominal Level	Achieved Missing (%)	Mean MAE	Mean ΔMAE (%)	Median ΔMAE (%)
A1	RMS/none	–	–	0.0330	+0.00	+0.00
A1	RMS/training jitter	–	–	0.0330	+0.09	+0.02
A1	Raw center sample/none	–	–	0.0394	+21.26	+13.18
A1	Raw center sample/training jitter	–	–	0.0395	+21.40	+13.46
A1	Spectral centroid/none	–	–	0.0379	+16.64	+14.03
A1	Spectral centroid/training jitter	–	–	0.0380	+16.75	+14.07
A2	Clean	0	0.00	0.0330	+0.00	+0.00
A2	Independent points	5	5.00	0.0339	+3.15	+3.45
A2	Independent points	10	9.97	0.0347	+6.06	+6.67
A2	Independent points	20	19.88	0.0363	+11.56	+13.24
A2	Blocks (length 4)	5	6.22	0.0334	+1.58	+1.73
A2	Blocks (length 4)	10	12.09	0.0339	+3.34	+3.67
A2	Blocks (length 4)	20	21.48	0.0349	+6.67	+7.19
A2	Blocks (length 16)	5/10	12.50	0.0338	+2.66	+2.92
A2	Blocks (length 16)	20	25.19	0.0349	+6.64	+7.66
A3	Clean	–	–	0.0330	+0.00	+0.00
A3	Raw-waveform AWGN	30 dB	–	0.0330	+0.00	+0.00
A3	Raw-waveform AWGN	20 dB	–	0.0330	+0.21	+0.13
A3	Raw-waveform AWGN	10 dB	–	0.0378	+15.70	+17.45

**Table 11:** DB2 compact ablation summary (vibration, H = 8). Nine forecasting methods are included. Corruption realizations are averaged within each model–seed cell before equal-weight aggregation across cells. Relative changes use RMS/no augmentation (A1) or clean input (A2–A3) as matched baselines. A2 reports the achieved rate; the nominal 5% and 10% block-16 cases are identical one-block masks and are collapsed.

Ablation	Setting	Nominal Level	Achieved Missing (%)	Mean MAE	Mean ΔMAE (%)	Median ΔMAE (%)
A1	RMS/none	–	–	0.06543	+0.00	+0.00
A1	RMS/training jitter	–	–	0.06528	−0.16	+0.00
A1	Raw center sample/none	–	–	0.11163	+128.33	+46.27
A1	Raw center sample/training jitter	–	–	0.11135	+128.04	+43.82
A1	Spectral centroid/none	–	–	0.09066	+82.56	+4.78
A1	Spectral centroid/training jitter	–	–	0.09054	+82.39	+4.86
A2	Clean	0	0.00	0.06543	+0.00	+0.00
A2	Independent points	5	4.94	0.06544	+0.03	+0.00
A2	Independent points	10	9.96	0.06548	+0.17	+0.00
A2	Independent points	20	19.84	0.06553	+0.34	+0.02
A2	Blocks (length 4)	5	6.22	0.06547	+0.14	+0.02
A2	Blocks (length 4)	10	12.09	0.06543	−0.04	+0.01
A2	Blocks (length 4)	20	21.49	0.06565	+0.73	+0.04
A2	Blocks (length 16)	5/10	12.50	0.06547	+0.06	+0.05
A2	Blocks (length 16)	20	25.19	0.06562	+0.44	+0.14
A3	Clean	–	–	0.06543	+0.00	+0.00
A3	Raw-waveform AWGN	30 dB	–	0.06542	−0.02	+0.00
A3	Raw-waveform AWGN	20 dB	–	0.06536	−0.17	−0.13
A3	Raw-waveform AWGN	10 dB	–	0.06505	−0.61	−0.02

## Data Availability

The core source code for preprocessing and model execution is publicly available under the MIT License at https://github.com/Thi-Thu-HuongLe/vibration-forecasting-code, accessed on 4 August 2026. DB1 is available from Mendeley Data at https://data.mendeley.com/datasets/ztmf3m7h5x/6, accessed on 17 May 2026 (DOI: 10.17632/ztmf3m7h5x.6). DB2 is available from NASA Open Data at https://data.nasa.gov/dataset/ims-bearings, accessed on 17 May 2026.

## Abbreviations

The following abbreviations are used in this paper:

ARIMA	Autoregressive Integrated Moving Average
AIC	Akaike Information Criterion
AWGN	Additive White Gaussian Noise
BPFI	Ball-Pass Frequency Inner-Race
BPFO	Ball-Pass Frequency Outer-Race
CI	Confidence Interval
CPU	Central Processing Unit
CUDA	Compute Unified Device Architecture
FP32	32-Bit Floating-Point Precision
GELU	Gaussian Error Linear Unit
GPU	Graphics Processing Unit
IMS	Intelligent Maintenance Systems
IQR	Interquartile Range
LSTM	Long Short-Term Memory
LS-SVM	Least-Squares Support Vector Machine
MAE	Mean Absolute Error
MASE	Mean Absolute Scaled Error
MSE	Mean Squared Error
NASA	National Aeronautics and Space Administration
NRMSE	Normalized Root Mean Square Error
PCA	Principal Component Analysis
PDM	Past-Decomposable-Mixing
PLC	Programmable Logic Controller
RevIN	Reversible Instance Normalization
ReLU	Rectified Linear Unit
RMS	Root Mean Square
RMSE	Root Mean Square Error
RSS	Resident Set Size
RUL	Remaining Useful Life
SD	Standard Deviation
SNR	Signal-to-Noise Ratio
sMAPE	Symmetric Mean Absolute Percentage Error
